# Taking Control: *Campylobacter jejuni* Binding to Fibronectin Sets the Stage for Cellular Adherence and Invasion

**DOI:** 10.3389/fmicb.2020.00564

**Published:** 2020-04-09

**Authors:** Michael E. Konkel, Prabhat K. Talukdar, Nicholas M. Negretti, Courtney M. Klappenbach

**Affiliations:** School of Molecular Biosciences, College of Veterinary Medicine, Washington State University, Pullman, WA, United States

**Keywords:** pathogenesis, bacteria-host cell interactions, adhesin, MSCRAMM, fibronectin

## Abstract

*Campylobacter jejuni*, a foodborne pathogen, is one of the most common bacterial causes of gastroenteritis in the world. Undercooked poultry, raw (unpasteurized) dairy products, untreated water, and contaminated produce are the most common sources associated with infection. *C. jejuni* establishes a niche in the gut by adhering to and invading epithelial cells, which results in diarrhea with blood and mucus in the stool. The process of colonization is mediated, in part, by surface-exposed molecules (adhesins) that bind directly to host cell ligands or the extracellular matrix (ECM) surrounding cells. In this review, we introduce the known and putative adhesins of the foodborne pathogen *C. jejuni.* We then focus our discussion on two *C. jejuni* Microbial Surface Components Recognizing Adhesive Matrix Molecule(s) (MSCRAMMs), termed CadF and FlpA, which have been demonstrated to contribute to *C. jejuni* colonization and pathogenesis. *In vitro* studies have determined that these two surface-exposed proteins bind to the ECM glycoprotein fibronectin (FN). *In vivo* studies have shown that *cadF* and *flpA* mutants exhibit impaired colonization of chickens compared to the wild-type strain. Additional studies have revealed that CadF and FlpA stimulate epithelial cell signaling pathways necessary for cell invasion. Interestingly, CadF and FlpA have distinct FN-binding domains, suggesting that the functions of these proteins are non-redundant. In summary, the binding of FN by *C. jejuni* CadF and FlpA adhesins has been demonstrated to contribute to adherence, invasion, and cell signaling.

## Recognition of *C. jejuni* as a Significant Foodborne Pathogen

*Campylobacter jejuni* has emerged from obscurity to become a leading bacterial cause of diarrheal disease over the course of five decades (Kaakoush et al., 2015). It was not until 1963 that Sebald and Véron proposed the term campylobacter (in Greek, a ‘curved rod’) to distinguish theses microaerophilic vibrios from the vibrios associated with cholera and other halophiles (Sebald and Véron, 1963). Although veterinarians were the first to recognize that this organism caused mild dysentery in cattle and sheep, a major breakthrough occurred in 1968 when *C. jejuni* was isolated from the diarrheal stool of a young adult using a special filtration technique (Dekeyser et al., 1972). This led to the development of a selective medium for *Campylobacter* isolation from diarrheal stools of both animals and humans, and a more accurate assessment of the public health burden of *C. jejuni* infections (Butzler and Skirrow, 1979). Presently, there are an estimated 1.3 million illnesses each year from *C. jejuni* in the United States and an estimated 96 million cases worldwide annually (Asuming-Bediako et al., 2019). In high-income countries, acute campylobacteriosis is characterized by fever, severe abdominal cramps, and diarrhea containing blood and leukocytes (Blaser et al., 1979; Karmali and Fleming, 1979; Svedhem and Kaijser, 1980). The most common source of *C. jejuni* infection is the handling or consumption of raw or undercooked poultry products, as chickens are the natural reservoir of this bacterium (Friedman et al., 2004). However, unpasteurized milk, eggs, untreated water, contaminated produce, and contact with animals colonized with *C. jejuni* have also been implicated as sources of infection (Horrocks et al., 2009; Bronowski et al., 2014; Huang et al., 2015). The economic impact of *C. jejuni* infections extends beyond the treatment of acute diarrheal illness, as infection with certain *C. jejuni* strains is correlated with a higher incidence of Guillain-Barré syndrome (GBS). GBS, an autoimmune syndrome, is the leading cause of flaccid paralysis in the post-polio era (Schwerer, 2002). Also, reports of antibiotic-resistant *Campylobacter* have continued to increase over time for multiple classes of antibiotics (Engberg et al., 2001; Coker et al., 2002; Bae et al., 2005; Gibreel and Taylor, 2006; Ruiz-Palacios, 2007). Recently, the Centers for Disease Control and Prevention listed drug-resistant *C*. *jejuni* as a ‘serious threat’^1^. Overall, *C. jejuni* has emerged as a pathogen of significance in human health due to the number of infections worldwide, the emergence of antibiotic-resistant isolates, and the bacterium’s association with post-infection sequelae. Given these factors, current efforts to determine the underlying mechanisms by which *C. jejuni* coordinates virulence during its interactions with host tissues should be expanded to develop new intervention strategies to reduce the global impact of campylobacteriosis. Among the most intensely studied *C. jejuni* virulence factors to date are host cell-binding proteins. Here we introduce the *C. jejuni* cell-binding proteins and then focus on the CadF and FlpA proteins, as these are the best-characterized adhesins.

## Adhesins Are Key Players at the Bacteria-Host Cell Interface: *C. jejuni* Adhesive Molecules

Bacteria have evolved an abundance of mechanisms to engage and alter the behavior of host cells. Some of these events are facilitated by hydrophobic interactions resulting in non-specific adhesion, while others are highly specific and dependent upon the binding of a bacterial molecule to a host surface receptor and/or component of the extracellular matrix (ECM) (Stones and Krachler, 2016). A common theme shared among pathogenic bacteria is the presentation of surface-exposed molecules known as adhesins. In this review, the term adhesin is defined as a bacterial molecule(s) that facilitates a specific interaction between an individual bacterium and a eukaryotic cell protein, glycoprotein, or glycolipid that is surface-exposed. Adhesins are highly specialized surface adhesive structures, which are comprised of single monomeric proteins as well as intricate multimeric macromolecules, that can play a crucial role in allowing bacteria to colonize and persist in a host (Pizarro-Cerda and Cossart, 2006). The advantage of a gut bacterium adhering to the host cells is that it can aid in establishing intestinal persistence, as the gastric fluids that bathe the surfaces of tissues, combined with the involuntary rhythmic contractions of peristalsis, may wash away bacteria. The specificity of adhesin:receptor interactions can take on many forms and may change over the course of an infection to enable the pathogen to target different host cells and tissues (tissue tropism). An adhesin may target one host cell molecule or may have several domains that permit binding to multiple surface receptors. Alternatively, multiple adhesins may work in an additive or cooperative manner to enable the bacterium to bind to one surface receptor. Adhesins may also be synthesized at different phases during infection in response to physicochemical properties. The colonization of host tissues can affect the gene expression of virulence-specific genes in bacteria as well as alter the bacterium’s metabolism and respiration. In several instances, bacterial cellular adherence is also used as a platform for type III, type IV, or type VI contact-dependent secretion systems to deliver effectors (virulence factors) that rewire host cell signaling pathways and behavior in dramatic fashion (Tsang et al., 2010; Stones and Krachler, 2016). Similar to other intensely studied bacteria, such as *Staphylococcus aureus* and *Salmonella* spp. (Vaca et al., 2019), the foodborne pathogen *C. jejuni* synthesizes several adhesins to promote binding to host cells. These adhesins are among the first molecules to make physical contact with a host cell. In addition, the FlpA adhesin, and presumably the CadF adhesin, permit the delivery of effector proteins into the cytosol of a host target cell (Larson et al., 2013).

*Campylobacter jejuni* binding to the cells lining the intestinal epithelium is dependent on multiple factors, including motility, bacterial cell surface charge, and multiple adhesins. Some 30 years ago, *C. jejuni* was thought not to synthesize specific adhesive proteins or specifically adhere to any tissues, but that host factors such as the O_2_/CO_2_ concentration and nutrient availability dictated the site of *C. jejuni* colonization in a host. However, in the late 1980’s and early 1990’s, *Campylobacter* researchers identified several proteins and molecules that could bind to host cells or host cell ligands, providing renewed energy for researchers to search for additional adhesins. While this age of discovery for new virulence determinants was an exciting time for *Campylobacter* researchers, additional studies raised questions regarding whether some of the identified proteins truly act as bacterial adhesins. For example, Cj0268c, Cj0289c (PEB3), Cj0596 (PEB4), Cj0921c (PEB1) are localized in the bacterial periplasmic space, and Cj1349c appears to be localized in the bacterial cytosol (Supplementary Table S1). Moreover, a few of these proteins were determined to have other functions (Leon-Kempis Mdel et al., 2006; Min et al., 2009; Kale et al., 2011). These facts highlight the need for more data to clearly demonstrate that an individual protein is surface exposed and binds to a host cell receptor/ligand. Additional research involving the use of a deletion mutant and a complemented isolate is also necessary to determine if a protein facilitates binding to a host cell ligand. The bacterial flagellum, capsular polysaccharide, and lipopolysaccharide have been reported to contribute to *C. jejuni* binding to cells, but the precise role of these bacterial structures in cellular adherence remains to be elucidated (Supplementary Table S2). Moreover, generating a deletion of one component of the structure often affects a cellular function (e.g., motility) or influences another bacterial property (e.g., surface charge). For example, deletion in the gene encoding the FlaA filament protein renders the bacteria non-motile. In the past forty years of research on *Campylobacter* virulence factors, a cellular target has been identified for three *C. jejuni* proteins; Cj0983 (JlpA) binds to HSP90, Cj1279c (FlpA) binds to fibronectin (FN), and Cj1478c (CadF) binds to FN (Supplementary Table S1). To gain further insight into other *C. jejuni* adhesins and their potential role in colonization and disease, we refer the reader to two previously published review articles (Rubinchik et al., 2012; Lugert et al., 2015).

**FIGURE 1:**
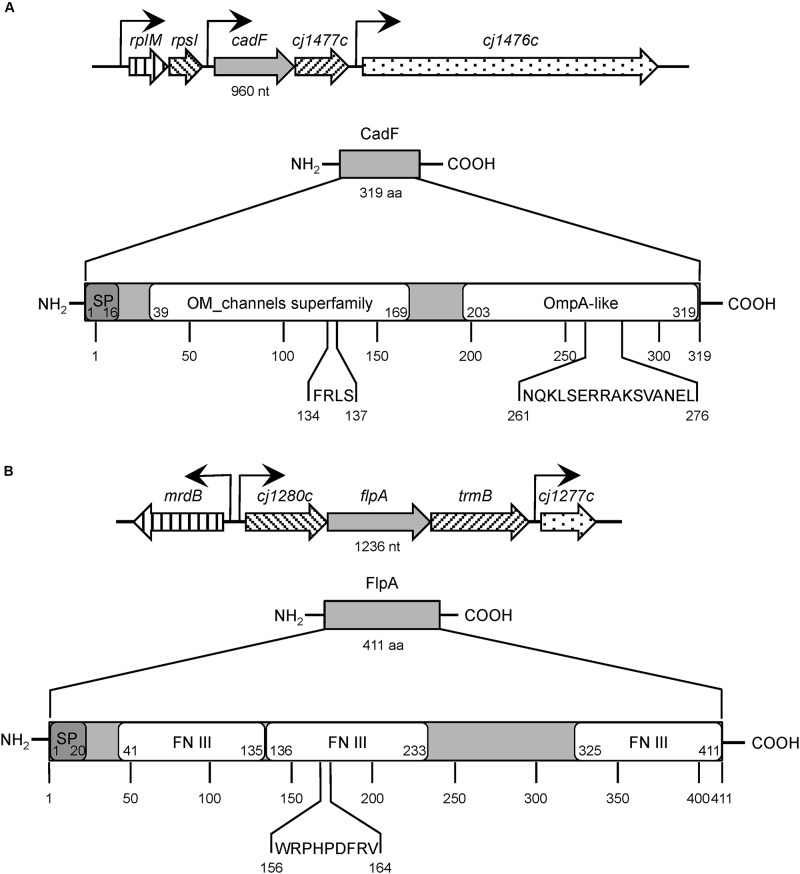
Structural organization of *Campylobacter jejuni* CadF and FlpA fibronectin-binding proteins. Panel **(A)** *C. jejuni* CadF, a 319 amino-acid protein, is encoded by the *cadF* gene, 960 nucleotides in size, expressed as the first gene of a bicistronic operon. CadF has two dominant protein domains: an outer membrane (OM) channel superfamily and an OmpA-like domain. The four amino-acid residues (FRLS) of fibronectin (FN)-binding motif are located in the OM channel superfamily domain at position 134 through 137. The C-terminal region of CadF also contains the 16-residue peptidoglycan-binding motif (NQKLSERRAKSVANEL) located in the OmpA-like domain at position 261 through 276. Panel **(B)** The *C. jejuni* FlpA protein is comprised of 411 amino-acid residues and is encoded by the *flpA* gene. The *flpA* gene is 1,236 nucleotides in size and is expressed as the second gene of a tri-cistronic operon. The FlpA protein has three individual FN type III-like (FN III) domains. The FN-binding motif of FlpA is nine amino-acid residues long (WRPHPDFRV) and located in the second FN III domain at position 156 through 164. The signal peptide sequences, which were identified using SignalP 5.0, are indicated with the ‘SP’ for both (**A** and **B**). The abbreviations ‘nt’ and ‘aa’ refers to nucleotides and amino acids, respectively. The gene information was obtained from the NCBI GenBank database; Accession No: AL111168.1. The protein structure domains were obtained from the UniProt database; CadF UniProtKB Accession No: Q0P8D9, FlpA UniProtKB Accession No: Q0P8X7.

Microbial Surface Components Recognizing Adhesive Matrix Molecule(s) (MSCRAMMs), which are synthesized by many pathogenic Gram-positive and Gram-negative bacteria, have been demonstrated to contribute to the disease process. Fibronectin-binding proteins (FNBPs) are members of the MSCRAMM family. This article focuses on the *C. jejuni* CadF and FlpA FNBPs, two adhesins that facilitate bacterial colonization and contribute to illness in a disease model (Figure 1, and Table 1). Continued investigation of the *C. jejuni* FNBPs is needed to fully understand the specificity of the CadF and FlpA proteins in mediating host cell interactions and to better compare these two proteins to the FNBPs of Gram-positive pathogens, Gram-negative pathogens, and commensal organisms.

**TABLE 1:** *Campylobacter jejuni* CadF and FlpA fibronectin (FN)-binding proteins (FNBPs).

**Property or characteristic**	**CadF (*Campylobacter* adhesion to Fibronectin)**	**FlpA (Fibronectin-like protein A)**
Gene organization	Predicted to be the first gene of a bicistronic operon (*cadF*, *Cj1477c*)	Predicted to be the second gene in an operon containing three genes (*Cj1280c*, *flpA*, and *Cj1278c*)

No. of nucleotides, residues, *M*_*r*_ (strain NCTC 11168)	960 nts, 319 aa, 37 kDa	1,236 nts, 411 aa, 46 kDa

Prominent features	Signal peptide of 16 residues in length (Konkel et al., 1997) and a consensus peptidoglycan-binding motif NX_2_LSX_2_RAX_2_VX_3_l (von Heijne, 1985) Fractionates in the outer membrane preparations and has been demonstrated to be surface-exposed using a rabbit CadF-specific serum (Konkel et al., 1997)	Lipoprotein signal sequence of 20 residues comprised of a tripartite structure and an invariant cysteine after the carboxy-terminus of the signal (Konkel et al., 2010) Deduced amino acid sequence contains three FN type III-like domains (Konkel et al., 2010) Fractionates in the outer membrane preparations and has been demonstrated to be surface-exposed using a rabbit FlpA-specific serum (Konkel et al., 2010)

Host cell target	FN	FN

FN-binding domain	Purified CadF displays dose-dependent and saturable FN-binding activity (Konkel et al., 2010) FN-binding domain has been localized to four amino acids [AA 134–137, Phe-Arg-Leu-Ser (FRLS)] (Konkel et al., 2005) FN-binding to an FRLS containing peptide is saturable (Konkel et al., 2005) FRLS domain determined to be surface-exposed using a mouse α-CadF peptide polyclonal antibody (Konkel et al., 2005) A rCadF protein containing the Ala-Ala-Gly-Ser residues at AA 134–137 exhibited a decrease in FN-binding (Konkel et al., 2005)	Purified FlpA protein displays dose-dependent and saturable FN-binding activity (Konkel et al., 2010; Larson et al., 2013) FN-binding domain has been localized to nine amino acids [AA 156–164, Trp-Arg-Pro-His-Pro-Asp-Phe-Arg-Val (WRPHPDFRV)] (Larson et al., 2013)

Site of binding to FN	The site of CadF binding on FN is not known	FlpA exhibits dose-dependent and saturable binding to the 40 kDa gelatin-binding domain of FN (Larson et al., 2013)

Adhesion to cells	A *cadF* mutant demonstrates reduced adhesion to human INT 407 epithelial cells (Monteville et al., 2003) The binding of *C. jejuni* to INT 407 cells is reduced by peptides containing the FRLS residues (Konkel et al., 2005) Competitive inhibition assays revealed that a *cadF* mutant inefficiently competes against the *C. jejuni* wild-type strain for binding to INT 407 cells compared to another wild-type strain (Monteville et al., 2003) Phenotypic changes were initially confirmed by studies performed with a *Cj1477c* mutant (Monteville et al., 2003)	A *flpA* deletion mutant demonstrates reduced adhesion to human INT 407 epithelial cells (Konkel et al., 2010) and chicken LMH hepatocellular carcinoma epithelial cells (Flanagan et al., 2009) Phenotypic changes were confirmed by in *trans* complementation studies (Konkel et al., 2010)

Additional cell assays	FN-facilitated invasion of T84 eukaryotic cells by *C. jejuni* occurs preferentially at the basolateral cell surface (Monteville and Konkel, 2002)	A polyclonal serum against FlpA blocks *C. jejuni* adherence to INT 407 cells in a concentration-dependent manner (Konkel et al., 2010)

Host cell signaling pathways	Phosphorylation of paxillin is reduced with a *C. jejuni cadF* insertional mutant (Monteville and Konkel, 2002) Rac1 and Cdc42 GTPase dependent cell-signaling events are blunted with a *C. jejuni cadF* mutant (Krause-Gruszczynska et al., 2007) A *C. jejuni cadF flpA* double mutant is impaired in the activation of the epidermal growth factor receptor and Rho GTPase Rac1 (Larson et al., 2013)	FlpA is required for phosphorylation of Erk1/2 during *C. jejuni* infection (Larson et al., 2013) A *C. jejuni cadF flpA* double mutant is impaired in the activation of epidermal growth factor receptor and Rho GTPase Rac1 (Larson et al., 2013)

Infection of chickens	A *cadF* insertional mutant is impaired in chicken colonization (Ziprin et al., 1999; Flanagan et al., 2009)	A *flpA* deletion mutant is impaired in chicken colonization – 2 of 10 chickens were colonized (Flanagan et al., 2009)

Vaccination of chickens	Vaccination results in a reduction in the median level of *C. jejuni* cecal colonization when compared to the *C. jejuni*-inoculated, non-vaccinated control group (Neal-McKinney et al., 2014)	Vaccination results in a reduction in the median level of *C. jejuni* cecal colonization when compared to the *C. jejuni*-inoculated, non-vaccinated control group (Neal-McKinney et al., 2014)

Human antibody response	Individuals infected with *C. jejuni* generate antibodies against CadF (Konkel et al., 1997)	Not yet tested

Other information related to potential disease	Abiotic IL-10^–/–^ mice show a reduced median pathology score for the *cadF* mutant and lower levels of TNF-α and IFN-γ compared to the wild-type strain (Schmidt et al., 2019) Undergoes post-translational processing to form smaller proteins of 24 kDa (CadF_24_) and 22 kDa (CadF_22_) that retain FN binding but that loses immunogenicity (Scott et al., 2010)	FlpA is required for *C. jejuni* dissemination to the spleen in IL-10^–/–^ germ-free mice (Larson et al., 2013)

## Fibronectin: a Multidomain Glycoprotein

Many bacterial adhesins target and bind to FN, which is ubiquitously present in the ECM of a variety of human tissues and organs, including intestinal epithelial cells (Frantz et al., 2010). Mature FN is a large glycoprotein (220 kDa) and can exist as two forms. Plasma FN is synthesized by hepatocytes and secreted into the blood plasma, saliva, and other body fluids, where it circulates as a compact dimer (To and Midwood, 2011). In contrast, cellular FN is synthesized and secreted by many cell types, including fibroblasts and endothelial cells, and becomes incorporated into the ECM to form a fibrillar-type (insoluble) matrix (Sottile and Hocking, 2002; To and Midwood, 2011). One of the stages of matrix assembly is FN unfolding, where cryptic binding sites are exposed (Smith et al., 2007; Weinberg et al., 2017). The unfolding of FN molecules enhances its binding to host cells, especially involving the β_1_ integrin subunit (Henderson et al., 2011). In mammals, there are 18 α and 8 β integrin subunits that can associate to form 24 integrin heterodimers, which are transmembrane ECM-binding proteins. The binding of FN to the α_5_β_1_ integrin heterodimer induces integrin clustering and promotes fibrillar assembly. Moreover, a tripartite linkage is established between FN, the integrins, and the actin cytoskeleton, allowing the cytoplasmic domains of the integrins to trigger intracellular signaling (Singh et al., 2010). FN incorporation into the cellular matrix has been demonstrated to play an important role in cell adhesion, cell migration, cell signaling, ECM remodeling, and tissue regeneration (To and Midwood, 2011).

Plasma (soluble) and cellular (insoluble) FN arise from alternative splicing of a single pre-mRNA (a single gene) (Pankov and Yamada, 2002). While there are variations in the composition of cellular and plasma FN, the proceeding discussion focuses primarily on cellular FN found in the ECM of tissues. The gene encoding cellular FN harbors Extra Domains A and B (EDA and EDB, also termed EIIIA and EIIIB) and a segment connecting two type III repeats (see below) called the type III connecting segment (IIICS, also termed the variable, or V, region). The alternative splicing of the EDA, EDB, and IIICS elements during transcription allows for the expression of different cellular FN isoforms (Schwarzbauer et al., 1983). In contrast to cellular FN, plasma FN does not harbor the EDA and EDB domains, and only one FN subunit possesses a IIICS segment. Typically, both plasma and cellular FN consist of three types of repetitive domains: 12 FN type I repeats (FN I), 2 FN type II repeats (FN II), and 15-18 FN type III repeats (FN III) (Pankov and Yamada, 2002). FN I and FN II are structurally rigid. In contrast, the FN III repeats are flexible due to the absence of disulfide bonds and are required for FN polymerization (Potts and Campbell, 1994). The multimodular structure and intermodular regions of FN provide the molecule with the ability to interact with other host proteins, including collagen, laminin, integrins, and fibrin. Moreover, the complexity of the FN molecule provides multiple targets for FNBPs to bind to this molecule (White et al., 2008).

## Bacterial Binding to Fibronectin

Pathogenic bacteria that have FNBPs can use them to promote tissue adherence and interface with host cell signaling complexes (Stones and Krachler, 2015). More specifically, a three-component bridge is formed between the FNBPs, host cell-associated FN, and transmembrane integrins (Hymes and Klaenhammer, 2016). The linkage between the bacteria and FN stimulates integrin occupancy and clustering, triggering the recruitment of cell signaling molecules and rearrangement of the host cell actin cytoskeleton (Wu, 2007; Zaidel-Bar et al., 2007; Deakin and Turner, 2008). In some instances, it is the intimate binding mediated by FNBPs that enables host cell invasion and intracellular multiplication, resulting in acute disease (Hymes and Klaenhammer, 2016).

In general, FNBPs contain an N-terminal signal sequence, a central binding region, and a C-terminal cell wall anchor (LPXTG motif). The very first observation that a bacterium, *S. aureus*, was capable of binding to FN was reported in 1978 (Kuusela, 1978). More specifically, the FNBPA and FNBPB proteins from *S. aureus* have been reported to bind to the heparin-binding domain of FN (Bae and Schneewind, 2003; Schwarz-Linek et al., 2003) and that the deletion of both genes is required to abolish the organism’s ability to bind FN (Greene et al., 1995). Since the initial discovery of an FNBP in *S. aureus*, at least 100 other FNBPs have been identified in both Gram-positive and Gram-negative bacteria (Henderson et al., 2011). Although many of these proteins have been found to possess distinct FN-binding domains, even more interesting is the fact that they bind to different regions within the FN molecule. For example, *S. aureus* FNBPA and FNBPB only bind to the N-terminal five-module region (FN I_1__–__5_, heparin-binding domain), whereas the *Streptococcus pyogenes* F1 and Sfb1 bind to the FN I_1__–__5_ region as well as the FN I_6__–__9_ gelatin-binding domain (Sela et al., 1993; Bae and Schneewind, 2003; Schwarz-Linek et al., 2003; Schwarz-Linek et al., 2004). Other FNBPs target different FN regions. For example, *Borrelia burgdorferi* BBK32 binds to multiple sites, including FN I_2__–__3_ of the heparin-binding domain, FN I_8__–__9_ of the gelatin-binding domain, and FN III_1__–__3_ (Kim et al., 2004; Harris et al., 2014). The binding of these FNBPs to different sites within the FN molecule likely has different biological consequences.

## CadF Is a *C. jejuni* Outer Membrane Protein Containing Fibronectin-Binding Residues

In the 1980-90s, studies were conducted to determine if *C. jejuni* were able to bind to various components of the ECM, including FN, collagen, laminin, and vitronectin (Kuusela et al., 1989; Konkel et al., 1997; Moser et al., 1997). Collagen is the most abundant protein found in the ECM and provides structural support to resident cells. Laminin is a major component of the basal lamina, influencing cell differentiation, migration, and adhesion. Vitronectin binds to the α_5_β_3_ integrin, promoting cell adhesion and spreading. Kuusela et al. (1989) were the first to report that *C. jejuni* isolates were able to bind to FN, and also reported that some isolates were able to bind to Type I, III, and V collagens. Several years later, the observation was made that *C. jejuni* bound to host cell retraction fibers, which are fingerlike projections enriched in ECM components. This prompted researchers to further investigate *C. jejuni* binding to various ECM components. In contrast to laminin and vitronectin, *C. jejuni* bound to coverslips that were treated with FN (FN-coated coverslips). Radioactive FN ([^125^I]-FN) was then used to probe blots of *C. jejuni* outer membrane protein (OMP) extracts and was found to specifically bind to a 37 kDa OMP (CadF) (Konkel et al., 1997). A rabbit anti-serum was generated against the 37 kDa protein and used to screen *Campylobacter* genomic phage expression libraries. This led to the identification of *Cj1478c*, designated *cadF* for *Campylobacter* adhesion to Fibronectin (Konkel et al., 1997). The *cadF* gene encodes a protein of 326 amino acids, with a calculated molecular mass of 36,872 Da (Figure 1).

The *C. jejuni* CadF protein is a surface-exposed OMP that binds to soluble and insoluble FN (Table 1). Key features of the deduced amino acid sequence included a signal peptide of 16 residues (von Heijne, 1985) and a consensus peptidoglycan-binding motif, NX_2_LSX_2_RAX_2_VX_3_l. Sequence analysis showed that the protein consists of an N-terminal transmembrane domain that forms a β-barrel pore and a C-terminal domain forming a mixed α/β fold. Additional evidence confirmed that the 37 kDa protein bound to FN. First, biotinylated FN bound to a protein with an apparent molecular mass of 37 kDa (CadF) in the *C. jejuni* OMP extracts as judged by ligand-binding blots (Konkel et al., 1997). Second, the FN-binding domain within CadF was localized to four amino acids [AA 134–137, Phe-Arg-Leu-Ser (FRLS)] using overlapping 30-mer and 16-mer peptides coupled with enzyme-linked immunosorbent assays (ELISA) (Konkel et al., 2005). Third, *C. jejuni* mutants containing insertions in *cadF*, which disrupted the coding sequence, demonstrated a significant reduction in binding to FN. Moreover, the FRLS domain of CadF was determined to be surface-exposed using a mouse polyclonal α-CadF peptide antibody coupled with laser scanning confocal microscopy (Konkel et al., 2005). Finally, a recombinant CadF protein containing mutated FRLS residues (AA 134-137, FRLS > AAGS) exhibited a 91% decrease in FN-binding activity compared to the unmodified/native CadF protein. Although the FN-binding residues within CadF have been identified, researchers have yet to identify the CadF-binding site in FN.

## FlpA Is a *C. jejuni* Outer Membrane Lipoprotein Containing Fibronectin-Binding Residues

A second FNBP was identified in 2009 and was designated FlpA for Fibronectin-like protein A (Flanagan et al., 2009). The *C. jejuni flpA* gene is 1,236 nucleotides (411 residues) and is capable of encoding a protein with a calculated molecular mass of 46,124 Da (Figure 1, Table 1). The *flpA* gene is located in an operon containing three genes (*Cj1280c*, *flpA*, and *Cj1278c*). Sequence analysis of FlpA revealed the presence of three domains with similarity to the FN type III domain. Based on the presence of the FN type III domains, assays were performed to determine if FlpA binds to FN and the FN-binding phenotype of a *C. jejuni flpA* mutant. Purified FlpA protein displays dose-dependent and saturable FN-binding activity, as judged by ELISA using purified FlpA-GST protein (Konkel et al., 2010). The FN-binding site within FlpA was localized to a span of nine amino acids: Trp-Arg-Pro-His-Pro-Asp-Phe-Arg-Val (Larson et al., 2013). Assays were also performed to determine where FlpA binds within the FN molecule; proteolysis of FN with thermolysin (protease, type X) is a well-documented method for generating fragments of 29, 40–45, 65, and 130 kDa. Interestingly, thermolysin-digested FN fragments retain their biological activity. FlpA was determined to bind to the 40–45 kDa fragment (gelatin-binding domain) composed of four FN I repeats (FN I_6__–__9_) and two FN II repeats (FN II_1_,_2_). FlpA is likely a surface-exposed lipoprotein based on the data obtained from analysis using SignalP 5.0 (Almagro Armenteros et al., 2019) coupled with the application of indirect immunofluorescence microscopy of *C. jejuni* incubated with the FlpA-specific serum (Konkel et al., 2010).

## Role of CadF AND FlpA in *C. jejuni* Colonization of Chickens

Campylobacteriosis often results from the handling and consumption of foods cross-contaminated with raw poultry products. The linkage between human infection and the handling of fresh poultry is mainly due to the fact that *C. jejuni* endemically colonizes commercial chicken flocks. Given that the antibodies passed from hens to chicks are partially protective against *Campylobacter* colonization, research has been performed to identify *C. jejuni* membrane-associated proteins recognized by maternal antibodies (Sahin et al., 2003). Immunoblots coupled with tandem mass spectrometry revealed a list of *C. jejuni* proteins recognized by maternal antibodies that included CadF and FlpA (Shoaf-Sweeney et al., 2008). This finding is consistent with previous findings that a *C. jejuni cadF* mutant and a *C. jejuni flpA* mutant are impaired in colonizing chickens (Ziprin et al., 1999; Flanagan et al., 2009). Based on the premise that specific adhesins are pivotal to colonization, disruption of *C. jejuni* adherence by anti-adhesin antibodies seems to be an obvious way to incapacitate the bacterium. In this regard, vaccination of chickens with a combination of CadF and FlpA peptides together with the full-length CadF and FlpA proteins resulted in an antibody response and a reduction in the median level of *C. jejuni* cecal colonization when compared to the *C. jejuni*-inoculated, non-vaccinated control group (Neal-McKinney et al., 2014). These results support the proposal that CadF and FlpA significantly contribute to *C. jejuni* chicken colonization.

Additional work is required to understand the interaction of *C. jejuni* within the gut of chickens and the biological consequences of *C. jejuni* infection in poultry (Awad et al., 2018). For decades, *C. jejuni* has been considered a commensal organism of poultry, as chickens, in contrast to humans, do not develop disease symptoms. More specifically, *C. jejuni*, which are principally found in the ceca (mucosal crypts) of chickens at very high CFUs, have been reported to stimulate a poor or inefficient inflammatory response, leading to tolerance and persistent cecal colonization (Hermans et al., 2012). In contrast, it has also been reported that certain *Campylobacter* strains can adversely affect the health and welfare of chickens and that the clinical signs of *C. jejuni* infection of poultry are not obvious (Humphrey et al., 2014). As our understanding of the bacteria and host cell factors that influence *C. jejuni* infection in chickens results in a better understanding of disease outcome, studies are warranted to determine if CadF and FlpA contribute to steps beyond the colonization of chickens.

## CadF AND FlpA Regulation in Response to Intestinal Conditions

The intestinal life cycle of *C. jejuni* requires transition to an animal’s gut, where it responds to changes in physiological conditions. The abundance of the CadF and FlpA proteins has been demonstrated to be responsive to host conditions, including temperature, oxygen levels, oxidative stress, and mucin (Tu et al., 2008; Hong et al., 2014; Koolman et al., 2016; Guccione et al., 2017; de Oliveira et al., 2019). For example, Hong and colleagues reported that culturing *C. jejuni* with porcine mucin resulted in an increase in 32 proteins and a decrease in 20 proteins compared to bacteria grown in the absence of mucin for 24 h using a label-free LC-MS/MS technique (Hong et al., 2014). In this study, more than a 3-fold increase was detected in CadF abundance after growth in medium supplemented with porcine mucin. In contrast, Tu et al. (2008) reported a 1.9-fold decrease in the expression of *cadF* when *C. jejuni* were cultured in medium with a component of human mucin (MUC2) for 12 h using quantitative RT-PCR. Based on this finding, the investigators concluded that the CadF protein is not required for the bacteria to penetrate the mucus barrier. Although the two investigations used different methodologies (Tu et al., 2008; Hong et al., 2014), it is possible that CadF levels change over the course of an infection. Furthermore, in a study conducted by Guccione et al., a 2.4-fold increase in CadF abundance and a 1.7-fold increase in FlpA abundance was reported following an oxygen-tension downshift (from high to low oxygen tension), as determined by label-free proteomic analysis (Guccione et al., 2017). Noteworthy is that the abundance of the Peb1A protein was also reduced in response to the shift from high to low oxygen-tension. The investigators rationalized their findings based on the primary functions of the proteins. Peb1A, originally proposed to be an adhesin, was subsequently found to be an ABC-transporter involved in aspartate and glutamate uptake whereas CadF and FlpA are dedicated adhesins. Although much work needs to be done to determine how *C. jejuni* responds to the host environment, the studies conducted to date suggest that this bacterium responds to the gut, in part, by increasing CadF and FlpA protein levels.

## *In Vitro* Evidence of Fibronectin Recognition by *C. jejuni* in Promoting Host Cell Adherence

Molecular biologists have utilized mutational studies to assess the function of a particular gene product. Likewise, a genetic approach has been taken to assess the individual role of CadF and FlpA in *C. jejuni*-host cell interactions by generating single-gene mutations. A *cadF* mutant was created by disrupting the *cadF* gene by homologous recombination via a single crossover event between the *cadF* gene on the chromosome and an internal fragment of the *cadF* gene on a suicide vector (Konkel et al., 1997). As mentioned above, the *C. jejuni cadF* knockouts were deficient in FN-binding. At the time, one of the obstacles facing *Campylobacter* researchers for the generation of a *cadF* complemented isolate was the fact that repeated attempts to clone the entire *cadF* gene and its endogenous promoter in *E. coli* failed, perhaps due to toxicity (Monteville et al., 2003; Mamelli et al., 2006). Because *cadF* and *Cj1477c* are the first and second genes of a bicistronic operon, the possibility was raised that *Cj1477c*, which encodes a putative hydrolase, might contribute to the observed reduction in host cell adherence. To address this concern, a knockout was generated in *Cj1477c*. While the *C. jejuni cadF* mutant was found to be deficient in binding to INT 407 cells (Monteville et al., 2003), no reduction was noted in the binding of the *Cj1477c* knockout to INT 407 cells when compared to a *C. jejuni* wild-type isolate. To address the contribution of the CadF adhesin in *C. jejuni*-host cell attachment in the context of other adhesive proteins, competitive inhibition adherence assays were performed with a *C. jejuni cadF* mutant and a *C. jejuni* wild-type strain and compared with a competition between two different *C. jejuni* wild-type strains. The adherence assay performed with the two wild-type strains revealed a dose-dependent decrease in the adherence of one strain when the inoculum of the competing wild-type strain was increased (Monteville et al., 2003). However, the *C. jejuni cadF* mutant was unable to competitively inhibit the binding of a *C. jejuni* wild-type strain to INT 407 cells (Monteville et al., 2003). The application of a polarized cell model revealed that *C. jejuni* translocate a cell monolayer via a paracellular route, and then bind to FN localized on the basolateral surface of cells via CadF (Monteville and Konkel, 2002). These findings were in accordance with earlier studies, indicating that CadF binds to host cell-associated FN and promotes *C. jejuni*-host cell adherence.

The generation of a *flpA* knockout and complemented isolate was more straightforward than for *cadF*, as *flpA* containing its endogenous promoter can readily be cloned in *E. coli*. The *flpA* deletion mutant was generated using standard molecular techniques, and the mutant isolate was complemented by the introduction of a shuttle vector harboring *flpA* driven by a constitutive promoter (in *trans* complementation). Adherence assays revealed that the binding of the *C. jejuni flpA* mutant to INT 407 epithelial cells was significantly reduced compared with that of a wild-type strain. The reduction in binding of the *C. jejuni flpA* mutant was judged to be specific since complementation of the mutant in *trans* with a wild-type copy of the gene restored the organism’s binding to INT 407 cells (Konkel et al., 2010; Larson et al., 2013). Moreover, rabbit polyclonal serum generated against FlpA blocked *C. jejuni* adherence to INT 407 cells in a concentration-dependent manner.

## *C. jejuni* CadF and FlpA Stimulate Host Cell Signaling Pathways Associated With Host Cell Invasion

**FIGURE 2:**
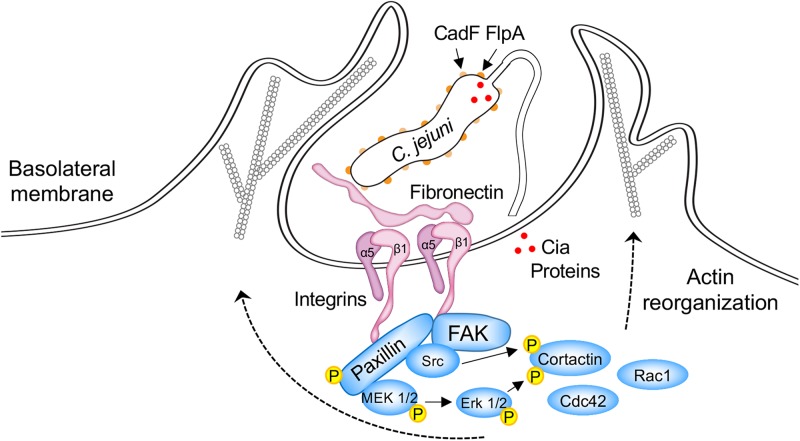
Schematic overview of host cell signaling events triggered by *C. jejuni* CadF and FlpA binding to fibronectin and engagement of the α_5_β_1_ integrin heterodimer. Focal adhesions (FAs) are dynamic protein complexes that connect the extracellular matrix to intracellular actin bundles. FAs contain over 100 proteins, including paxillin, focal adhesion kinase (FAK), and Src. Paxillin is a multi-domain adaptor protein that localizes to FAs. During FA assembly, paxillin becomes indirectly associated with the tails of β integrin subunits, is phosphorylated on multiple tyrosine, serine, and threonine residues, and serves as a central hub for other proteins. In particular, the phosphorylation of paxillin provides a binding platform for FAK and Src. The FAK-Src dual-activated signaling complex is responsible for the recruitment and activation of additional signaling and adaptor proteins that result in the activation of the Cdc42 and Rac1 Rho GTPases. Cortactin is a monomeric cytoplasmic protein that is also involved in polymerization and rearrangement of the actin cytoskeleton. The coordinated effort of key signaling proteins, scaffold proteins, and cytoskeletal components stimulate the formation of actin-based membrane protrusions and bacterial invasion. *C. jejuni cadF flpA* mutants are impaired in the ability to stimulate the activation of paxillin, Erk1/2, and Cdc42/Rac1 Rho GTPases (see the text for additional detail). The delivery of the Cia effector proteins (red dots) to the cytosol of host cells is impaired in a *C. jejuni flpA* mutant. Not known is whether CadF participates in the delivery of the effector proteins to host cells. The reader is referred to other papers for detailed models of *C. jejuni*-host cell interactions (Krause-Gruszczynska et al., 2007, 2011; Boehm et al., 2011; Eucker and Konkel, 2012; Konkel et al., 2013; Samuelson and Konkel, 2013).

A model of the *C. jejuni-*host cell interface is presented in Figure 2. This figure shows the interaction of the CadF and FlpA proteins with host cell-associated FN and α_5_β_1_ integrins, and the cell signaling pathways activated upon *C. jejuni* binding FN. *C. jejuni* manipulates focal adhesions (FAs) to stimulate host cell signaling and promote cell invasion (Eucker and Konkel, 2012; Konkel et al., 2013; Larson et al., 2013; Samuelson and Konkel, 2013). FAs are dynamic cellular structures that link ECM components, including FN, to intracellular cytoskeletal structures. They are comprised of integrin receptors, adaptor proteins, signaling proteins, and actin. Central to signaling through the FA is paxillin, which serves to integrate and disseminate signals from integrins to associated kinases that regulate membrane trafficking and cytoskeletal rearrangement (Schaller, 2001; Zaidel-Bar et al., 2007; Deakin and Turner, 2008). Although paxillin does not exhibit enzymatic activity, when phosphorylated, it serves as a central hub for other proteins. More specifically, phosphorylated paxillin acts as a scaffold for both signaling [kinases such as focal adhesion kinase (FAK) and Src] and adaptor proteins (vinculin and p130Cas). It is the establishment of the mature FA that ultimately results in signals being transduced from the integrins to the actin cytoskeleton (outside-in signaling).

CadF and FlpA appear to set the stage for cell invasion by promoting *C. jejuni* binding to the FN present on the basolateral surfaces of cells (i.e., *C. jejuni* bound to FN bound to the α_5_β_1_ integrin) and by initiating host cell signaling events. More specifically, *C. jejuni* infection of INT 407 epithelial cells results in the phosphorylation of paxillin, and the phosphorylation is partially dependent on CadF (Monteville et al., 2003). This is based on the findings that a lower Multiplicity of Infection (MOI) of the *C. jejuni cadF* mutant (100 to 1) did not induce phosphorylation of paxillin. However, paxillin phosphorylation was observed in cells infected with a higher MOI of the *C. jejuni cadF* mutant (2000 to 1). This implies that CadF is not solely responsible for the host cell signaling. Consistent with paxillin activation playing a role in *C. jejuni* invasion, treatment of epithelial cells with TAE 226 and PP2, selective inhibitors of FAK and Src kinase activity, results in a significant decrease in *C. jejuni* internalization as judged by the gentamicin-protection assay (Eucker and Konkel, 2012). *C. jejuni* internalization is also significantly reduced in the presence of both PD168393 and erlotinib, which are specific inhibitors of epidermal growth factor (EGF) receptor tyrosine phosphorylation (Eucker and Konkel, 2012). This is relevant because the EGF receptor can be stimulated in the absence of an extracellular ligand via integrin signaling, and the activation of this receptor can alter components of the cytoskeleton involved with actin organization, FA formation and resolution, and cell-cell adhesion (Thelemann et al., 2005). Researchers have reported that *C. jejuni* induces EGF receptor activation in a CadF and FlpA dependent manner; however, whether one or both proteins are required for EGF receptor activation is currently not known (Eucker and Konkel, 2012).

β_1_ integrins, FAK, Src, and paxillin also contribute to the activation of the MAP kinase Erk1/2 in *C. jejuni* infected cells (Eucker et al., 2014). Moreover, the phosphorylation of Erk1/2 is associated with FlpA-mediated activation of β_1_ integrins and EGF receptor signaling (Larson et al., 2013). Consistent with this finding, inhibition of Erk1/2 activation with PD98059 results in a significant reduction in *C*. *jejuni* internalization (Zaidel-Bar et al., 2007; Deakin and Turner, 2008). Finally, FAs can also activate the Rac1 and Cdc42 Rho GTPases, thereby stimulating the formation of actin-based membrane protrusions and bacterial invasion (von Heijne, 1985; Mitra et al., 2005; Hu et al., 2006; Krause-Gruszczynska et al., 2007; Eucker and Konkel, 2012; Neal-McKinney and Konkel, 2012; Samuelson and Konkel, 2013; Figure 2). Noteworthy is that GTPase dependent cell-signaling events are blunted with a *C. jejuni cadF* mutant compared to a wild-type strain (Krause-Gruszczynska et al., 2011; Boehm et al., 2012). Collectively, these findings demonstrate that *C. jejuni* binding to host cell-associated FN, via CadF and FlpA, is necessary to stimulate signaling pathways associated with cellular invasion. A potential caveat to these findings is that the binding of the *C. jejuni cadF* and *flpA* mutants to cells is reduced when compared to a wild-type strain, which could disrupt the delivery of secreted effector proteins.

## Delivery of *C. jejuni* Effector Proteins to Cells

Researchers have reported that the treatment of *C. jejuni* with chloramphenicol, a specific inhibitor of bacterial protein synthesis, significantly reduced host cell internalization, as compared to untreated controls (Konkel and Cieplak, 1992; Negretti et al., 2019). However, chloramphenicol treatment has little effect on *C. jejuni* adherence to host cells. Together, these data demonstrate that *C. jejuni* binding to host cell ligands is facilitated by adhesins, which are not sufficient to promote host cell internalization, but rather that internalization requires a second signal to stimulate host cell signaling pathways. The simplest view is that the *C. jejuni* FNBPs promote the bacterium’s binding to host cells, whereas *C. jejuni* invasion of epithelial cells requires *de novo* bacterial protein synthesis (McSweegan and Walker, 1986; Karlyshev and Wren, 2005). The question then arises of whether the *C. jejuni* CadF or FlpA proteins set the stage for cell invasion by merely binding to FN or whether they prime/stimulate host cell signaling pathways (albeit not to the level that would promote cell invasion).

The translocation of bacterial Type III secretion effectors to the cytosol of a host cell has been demonstrated using the adenylate cyclase domain (ACD) of *Bordetella pertussis* CyaA as a reporter (Sory and Cornelis, 1994; Desvaux et al., 2006; Ono et al., 2006; Schechter et al., 2006; Neal-McKinney and Konkel, 2012). The basis of the ACD assay is that adenylate cyclase (adenylyl cyclase) only catalyzes the production of cAMP when bound by calmodulin in the eukaryotic cell cytosol (Ladant and Ullmann, 1999; Dautin et al., 2002). *C. jejuni* was first reported to secrete proteins in 1999 (Konkel et al., 1999b) and later found to export proteins via the flagellar Type III Secretion System (T3SS) (Konkel et al., 2004). These secreted proteins were termed the *Campylobacter* invasion antigens (Cia), as they were found to contribute to cell invasion (Konkel et al., 1999b).

The delivery of most Type III secreted effectors requires the bacteria to contact the host cells (Schlumberger et al., 2005; Winnen et al., 2008). Different Cia proteins have been studied in several different *C. jejuni* strain backgrounds, including 81-176, NCTC 11168, and F38011. The Cia proteins have been found to play a role in *C. jejuni* cell invasion and survival but not in cell adherence (Christensen et al., 2009; Novik et al., 2010; Buelow et al., 2011; Samuelson et al., 2013; Naz, 2014). The most studied Cia proteins to date are CiaC and CiaD, which are necessary for maximal *C. jejuni* cellular invasion (Christensen et al., 2009; Neal-McKinney and Konkel, 2012; Samuelson and Konkel, 2013; Samuelson et al., 2013). *Cj1242* (*ciaC*) and *Cj0788* (*ciaD*) were initially identified to harbor a T3SS export signal using a genetic screen in a heterologous system, and *Cj0788* (*ciaD*) was also identified to play a role in *C. jejuni* cell invasion using a non-targeted transposon screen (Christensen et al., 2009; Novik et al., 2010). Relevant to the role of *C. jejuni* adhesins in Cia delivery to host cells was the discovery that the CiaC effector protein was delivered to the cytosol of host target cells using the ACD assay (Neal-McKinney and Konkel, 2012). Two additional experiments support the proposal that CiaC delivery requires bacteria-host cell contact. First, no increase was observed in intracellular cAMP levels when a bacterial supernatant containing the CiaC-ACD fusion protein was added to INT 407 cells versus non-infected cells. Second, no cAMP production was observed when a 0.2 μm pore filter was used to block the physical contact of *C. jejuni* with host cells (a two-chamber system: the apical chamber contained a *C. jejuni* strain harboring a *ciaC-ACD* construct and the bottom chamber contained the epithelial cells) (Neal-McKinney and Konkel, 2012). Similar methods were then used to test whether CiaC could be delivered to the host cell cytosol using a *C. jejuni flpA* mutant (Larson et al., 2013). The assay was performed in parallel with a *C. jejuni* wild-type strain transformed with the *ciaC-ACD* construct. Significantly less cAMP was detected in the cytosol of host cells infected with the *C. jejuni flpA* mutant when compared to cells inoculated with the *C. jejuni* wild-type strain transformed with the *ciaC-ACD* construct. Experiments have yet to be performed to determine if a Cia effector can be delivered to host cells from a *C. jejuni cadF* mutant. Taken together, it seems reasonable that the *C. jejuni* adhesins work in conjunction with the effector proteins to alter host cell behavior, including promoting host cell internalization.

## Potential Role of CadF and FlpA in Human Disease

Presumably, the ability of *C. jejuni* to bind to the cells lining the human gastrointestinal tract is necessary for disease. In one study, *C. jejuni* isolates recovered from individuals with fever and diarrhea were found to adhere to cultured cells at a greater efficiency than those strains isolated from individuals without diarrhea or fever (Fauchere et al., 1989). Whether the adherence capacity and disease severity are correlated for *C. jejuni* isolates needs to be further studied. Based on the data generated with *C. jejuni* infection of cultured human intestinal cells, it is probable that CadF and FlpA mediate *C. jejuni* binding to the epithelial cells lining the human intestinal tract. This binding is likely to set the stage for host cell invasion and intracellular multiplication, resulting in acute disease (van Spreeuwel et al., 1985; Black et al., 1988). Several options exist for how *C. jejuni* may gain access to FN as a substrate; three possibilities are discussed below. Each possibility is predicated on the belief that the bacteria must be able to penetrate the mucus overlying the intestinal epithelial cells and that FN is primarily found at the basolateral (internal) surface or between cells within the gut epithelium. First, *C. jejuni* could target sites where the host intestinal cells are shed by extrusion (at the tips of the intestinal villi), similar to that proposed for *Listeria monocytogenes* (Pentecost et al., 2006). Data supporting this possibility has been observed in *C. jejuni*-infected piglets, where the infection site is evident from villus blunting and cell necrosis (Babakhani et al., 1993). Piglets infected with different strains of *C. jejuni* develop a range of clinical symptoms similar to humans, ranging from watery stools to bloody diarrhea. The dysenteric-like illness (blood in the stool) is a severe form of the disease and is illustrative of cell adherence/invasion. Second, *C. jejuni* could reach the basolateral surfaces of the intestinal cells by cellular translocation. Laboratory studies with polarized intestinal cells have demonstrated that *C. jejuni* can translocate across an intact cell barrier by migrating between cells (i.e., a paracellular route of translocation) (Monteville and Konkel, 2002; Boehm et al., 2012). Third, *C. jejuni* may breach the intestinal barrier by transcytosis across Microfold (M) cells in Peyer’s patches of the intestine (Walker et al., 1988). Together, it is possible for the bacteria to either initially colonize at sites where the epithelium is disrupted and FN is readily accessible and/or bind to FN on the basolateral surface of epithelial cells following cellular translocation or via passage across Peyer’s patches.

Two animal studies have been done to date in order to specifically address the role of CadF and FlpA in disease (Larson et al., 2013; Schmidt et al., 2019). Recently, the contribution of CadF in clinical disease was assessed using an abiotic (gnotobiotic) IL-10^–/–^ mouse model (Schmidt et al., 2019). In this model, the mice were inoculated with 10^9^ CFU of a *C. jejuni* wild-type isolate and *cadF* mutant, on two consecutive days, and disease parameters were assessed daily for six days. While the authors concluded that CadF is not required for campylobacteriosis in mice, the pro-inflammatory responses, such as TNF-α and IFN-γ, were lower for the *cadF* mutant compared to the wild-type isolate. In addition, the median pathology score for the *cadF* mutant was approximately 8 at day six, whereas a median pathology score of 12 was recorded for the mice infected with the wild-type strain. Disease severity was determined based on the presence of blood in the stool, diarrhea, and animal behavior. Thus, the mice inoculated with the *cadF* mutant develop less severe disease symptoms when compared to mice inoculated with the wild-type strain. These data demonstrate that CadF, while not being essential for disease, contributes to the severity of disease in a mouse model. Because *C. jejuni*-cell adherence is multifactorial, we speculate that it is possible to overcome the involvement of CadF and FlpA in a disease model by using high or repeated doses of mutant bacteria. Regarding the role of the FlpA protein in disease, a study with IL-10^–/–^ germ-free mice reported a reduced number of the *C. jejuni flpA* mutant in spleen when compared to mice inoculated with the *C. jejuni* wild-type strain (Larson et al., 2013). Studies are required to determine whether CadF and FlpA are necessary for human disease.

Although CadF is highly immunogenic, Scott and colleagues published an article revealing the possibility that CadF could retain its adhesive property and escape immune recognition in a host by post-translational processing (Scott et al., 2010). More specifically, the investigators demonstrated that CadF undergoes post-translational processing (proteolytic cleavage) to form smaller proteins of 24 kDa (CadF_24_) and 22 kDa (CadF_22_). Interestingly, the CadF_24_ and CadF_22_ variant forms were fully capable of binding to FN but were not recognized by patient sera. In addition, the processing of CadF appeared to be less abundant in the NCTC 11168 laboratory-adapted or avirulent strain (“GS” genome sequenced strain) when compared to the NCTC 11168 virulent strain (“O” original strain). The investigators concluded that the processing of CadF to the CadF_24_ and CadF_22_ variants provided antigenic variation that enabled evasion from the host immune response while enabling the protein to retain its adhesin-like function. Based on these findings, studies are warranted to determine whether the post-translational processing of CadF is common in other *C. jejuni* strains. Taken together, the data support the proposal that CadF may play a role in *C. jejuni* pathogenesis in a human host.

## Presence of CadF and FlpA in *C. jejuni* Isolates

Previous survey studies have suggested that the *cadF* and *flpA* genes are conserved amongst *C. jejuni* isolates (Konkel et al., 1999a; Ripabelli et al., 2010; Ghorbanalizadgan et al., 2014; Levican et al., 2019; Wei et al., 2019). To explore this proposition, we assessed 20,218 full *C. jejuni* genome sequences that were available on the GenBank FTP server as of August 1st, 2019. After the removal of misidentified isolates (*Campylobacter coli, Campylobacter upsaliensis*, and *Campylobacter lari*), the remaining 20,166 sequences were searched for the presence of the *cadF* or *flpA* genes using the blastn command line tool with the default parameters. The *cadF* gene was absent in the genomes deposited for eight *C. jejuni* isolates, and the *flpA* gene was absent in the genomes for another seven isolates (Supplementary Figure S1). No genomes were identified in which both the *cadF* and *flpA* genes were absent. The caveat of this analysis is that the genomes reported for these 15 isolates may not be complete; it is not possible to determine the accuracy/completeness of the deposited sequences. At the very least, this analysis indicates that at a minimum, 99.93% of the sequenced *C. jejuni* isolates have both *cadF* and *flpA*. Based on this bioinformatic analysis, we conclude that *cadF* and *flpA* are ubiquitous and highly conserved amongst *C. jejuni* isolates.

Molecular-based diagnostics are being used more and more frequently in epidemiological studies. As opposed to conventional diagnostic methods utilizing a combination of culture and biochemical testing, molecular methods (e.g., polymerase chain reaction, PCR) offer the advantage of being fast. Relevant to this review is that *cadF* is a common target gene for the testing of both human and animal samples for *Campylobacter* spp. More specifically, epidemiologists have utilized *cadF* as a *C. jejuni*/*C. coli*-specific diagnostic marker. In contrast to other potential virulence-associated genes [e.g., the CDT subunit genes (*cdtA, cdtB*, and *cdtC*), *iamA*, *iamB*, and *vir*B11], *cadF* is present in nearly 100% of *C. jejuni* and *C. coli* isolates (Supplementary Figure S1) (Ghorbanalizadgan et al., 2014; Levican et al., 2019; Wei et al., 2019). The *cadF* and *flpA* genes do not appear to be present in other pathogenic bacteria. Given that molecular diagnostics is becoming a more common approach, we have cited only a few articles where researchers have used PCR amplification of the *cadF* gene for diagnostic purposes (Ghorbanalizadgan et al., 2014; Francois et al., 2018; Levican et al., 2019; Wei et al., 2019).

## Perspective (Summary and Future Directions)

MSCRAMMs are fascinating in that they help establish an infection for many Gram-positive and Gram-negative bacteria. While many pathogenic bacteria harbor MSCRAMMs, it is evident from several decades of research that *C. jejuni* is a unique pathogen. The identification of CadF and FlpA in *C. jejuni*, two distinctive MSCRAMMs, is a perfect illustration of this fact, as the FN-binding domains within CadF and FlpA are unique among FNBPs. Moreover, CadF and FlpA are two proteins that have drawn the attention of researchers due to their adhesive properties, as well as their potential role in stimulating host cell signaling pathways and cytoskeletal rearrangement (via a mechanism that is either direct or indirect).

The CadF and FlpA FNBPs from *C. jejuni* are currently recognized as important virulence factors, as they are involved in the early interactions occurring at the bacteria-host cell interface. Both CadF and FlpA bind to FN, facilitate *C. jejuni* adherence to host cells, and enable *C. jejuni* colonization of chickens at a high level. Also known are the FN-binding domains within both the CadF and FlpA proteins, as well as the FN fragment to which the FlpA protein binds. Experimental evidence suggests that *C. jejuni* binding to FN allows the microorganism to communicate with the cell cytoskeleton via outside-in signaling through integrins, leading to bacterial internalization into a host cell. Internalization by an intestinal cell allows the bacterium to avoid the powerful killing mechanisms of a phagocyte. Several studies suggest that *C. jejuni* invasion of the cells lining the intestinal tract is a primary mechanism of colonic damage (van Spreeuwel et al., 1985; Babakhani et al., 1993; Russell et al., 1993). While CadF and FlpA are required for maximal host cell invasion (Eucker and Konkel, 2012), the contribution of these two proteins to invasion is, in part, due to their role in triggering host cell signaling pathways, possibly by aiding the delivery of effector proteins. Relevant to this point is that CadF and FlpA are involved in the activation of Cdc42 and Rac1 in human cells (Krause-Gruszczynska et al., 2011; Boehm et al., 2012; Eucker and Konkel, 2012; Larson et al., 2013); these are the two primary host cell Rho GTPases involved in *C. jejuni* invasion. These findings are likely due to the fact that *C. jejuni* adhesion mutants, including a *flpA* mutant, are impaired in the delivery of effector proteins to host cells (Larson et al., 2013). In addition, we propose that CadF and FlpA are both involved in the activation of the β_1_ integrin, which is required for *C. jejuni* invasion (Krause-Gruszczynska et al., 2011; Konkel et al., 2013). Studies are needed to address outstanding questions (e.g., Where does CadF bind within the FN molecule?; Do CadF and FlpA bind to FN simultaneously or is binding sequential?; Do CadF and FlpA bind to other substrates or ECM components?; What role do CadF and FlpA play individually in binding to FN and initiating downstream host cell signaling events?; Do other *C. jejuni* adhesins work cooperatively with CadF and FlpA?; Are CadF and/or FlpA necessary for biofilm formation?, etc.).

In spite of CadF and FlpA having unique FN-binding domains, the mechanistic basis for how they modify FN function will likely have broader implications given the number of pathogenic microbes that have FNBPs. As mentioned earlier, the FN molecule is a complex glycoprotein composed of multiple repeating domains. The different domains in FN allow this glycoprotein to bind to cells and to molecules within the surrounding matrix simultaneously. Moreover, pathogen binding to specific domains may afford different functional outcomes (e.g., cell binding, cell invasion, etc.). While many FNBPs target the 29 kDa N-terminal fragment of FN that is composed of five FN I repeats (FN I_1__–__5_), other FNBPs bind to the 40–45 kDa FN fragment that is comprised of four FN I and two FN II repeats (FN I_6_ - FN II_1__–__2_ - FN I_7__–__9_). While FlpA has been determined to bind to the 40–45 kDa fragment, it is not known where CadF binds to the FN molecule. It is possible that the binding of a pathogen to a distinct FN domain can alter various FN functions, including the modulation of integrin function. It will be of interest to compare the functional attributes of the CadF and FlpA proteins from *C. jejuni* with the FNBPs from other more intensely studied bacterial pathogens, as this additional information may provide further insight into pathogenic mechanisms. Finally, continued investigation of FNBPs is necessary to provide a greater understanding of their diversity and specificity in mediating bacteria-host cell interactions. Please note that following the submission of this article, we submitted a research article on the cooperative interaction of CadF and FlpA in binding to FN and host cells that has been published (Talukdar et al., 2020).

## Author Contributions

MK  worked on the original draft preparation, Table 1 preparation, project design and management. PT reviewed the manuscript, prepared Figure 1 and Supplementary Tables S1, S2. NN reviewed the manuscript, prepared Figure 2 and Supplementary Figure S1. CK reviewed the manuscript and prepared Figure 2.

## Conflict of Interest

The authors declare that the research was conducted in the absence of any commercial or financial relationships that could be construed as a potential conflict of interest.

## Footnotes

1 https://www.cdc.gov/drugresistance/biggest_threats.html
