# Supplementary material for: Taking Control: Campylobacter jejuni Binding to Fibronectin Sets the Stage for Cellular Adherence and Invasion
Source: Front Microbiol. 2020 Apr 9;11:564. doi: 10.3389/fmicb.2020.00564 (PMC7161372; doi:10.3389/fmicb.2020.00564)
Supplement: Supplementary file 1 [file Data_Sheet_1.pdf]

Taking control: *Campylobacter jejuni* binding to fibronectin sets the stage for cellular adherence and invasion

Michael E. Konkel, Prabhat K. Talukdar, Nicholas M. Negretti, and Courtney M. Klappenbach

School of Molecular Biosciences, College of Veterinary Medicine, Washington State University, Pullman, WA, USA, 99164-7520

Running title: *C. jejuni* fibronectin-binding proteins

Keywords: Pathogenesis, bacteria-host cell interactions, adhesin, MSCRAMM, fibronectin

Supplemental Materials and Figures

Supplemental Figure 1. *Campylobacter jejuni* isolates lacking either the *cadF* gene or the *flpA* gene. A total of 20,218 *C. jejuni* genome sequences from the GenBank FTP server were analyzed for the presence of *cadF* and *flpA*. All 'full genomes' were downloaded from ftp://ftp.ncbi.nlm.nih.gov/genomes/genbank/bacteria/Campylobacter_jejuni/ on August 1st, 2019. The blastn command line tool (version 2.2.31+) was used with default parameters to search each genome. Panel A) 52 sequences were omitted from the inspection of the 20,218 sequences, as these isolates appeared to be misidentified as *C. jejuni* isolates (i.e., their genomic sequences were most similar to *Campylobacter coli, Campylobacter upsaliensis,* and *Campylobacter lari*). All genomes predicted to lack *cadF* or *flpA* were clustered using JolyTree using default parameters, and a figure produced with FigTree. The figure shows the four reference sequences (*C. jejuni* NCTC 11168 = red line, red text, *C. lari* RM2100 = blue text, *C. upsaliensis* DSM 5365 = magenta text, and *C. coli* OR 12 = green text), the eight *C. jejuni* isolates lacking *cadF* (red lines, blue circles), the seven *C. jejuni* isolates lacking *flpA* (red lines, green circles), and the 52 misidentified genome sequences (black lines, black text). All isolates are labeled with their NCBI Biosample ID number. The tree is rooted at the midpoint. Branch support statistics are indicated at each fork with a length greater than 0.003, and the scale bar indicates the number of nucleotide substitutions per position. Panel B) The 20,166 genome sequences were subjected to analysis by blastn. Pertinent information for the eight *C. jejuni* isolates missing *cadF* and the seven *C. jejuni* isolates missing *flpA* is indicated in the table. *C. jejuni* isolates lacking both *cadF* and *flpA* genes were not found.

A

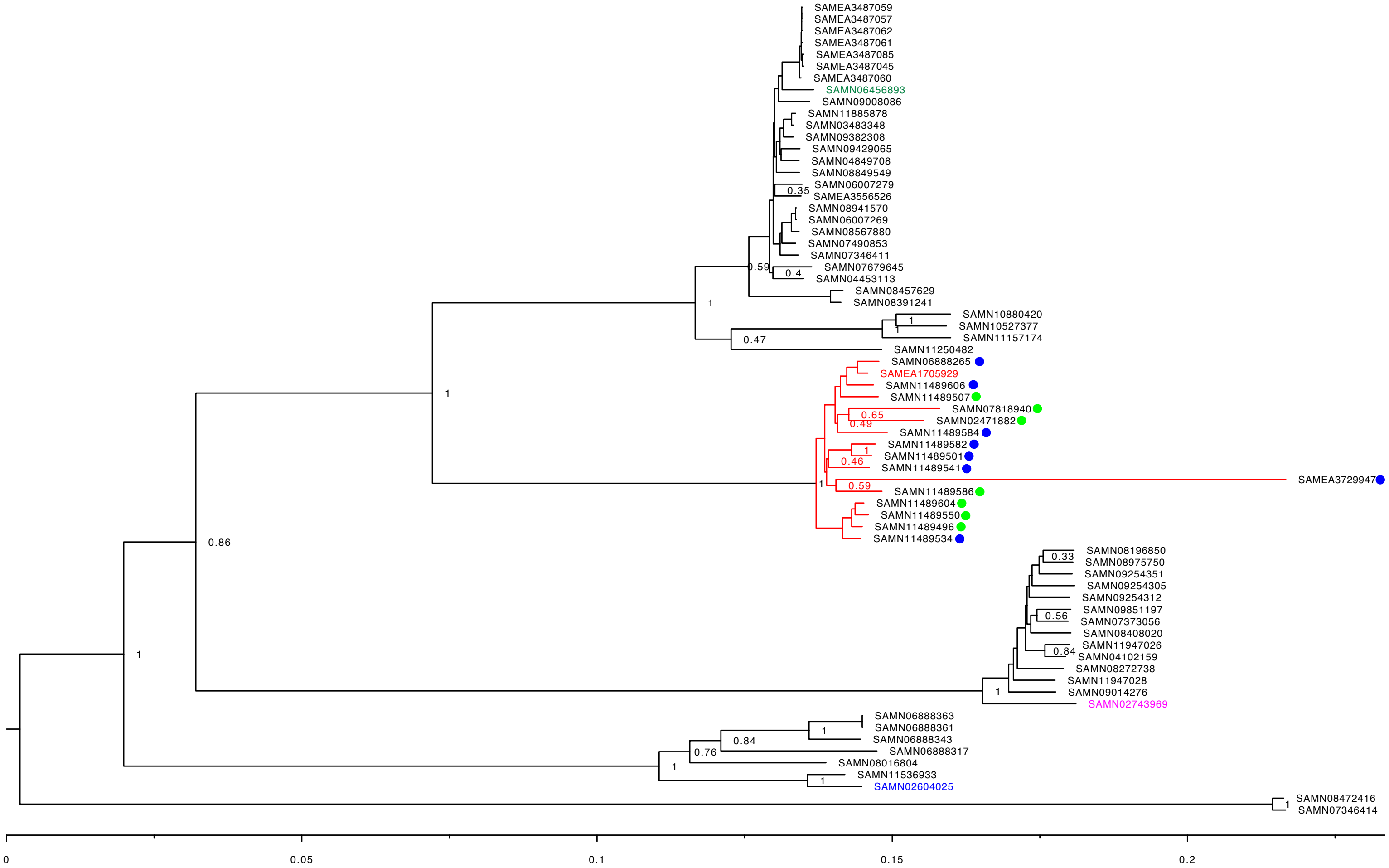

B

Missing *cadF*

Bioproject	Biosample	Organismn name	Infraspecific name	Seq release date	Submitter	Host	Isolation source	Collection date	Geographic location	Core genome sequence type
PRJNA312235	SAMN06888265	Campylobacter jejuni	strain=isolate_B3	6/8/17	Swansea University	Bos taurus	feces	2005	Canada	cgST-15704
PRJNA534408	SAMN11489606	Campylobacter jejuni	strain=PHL178	6/14/19	HEALTH SERVICE EXECUTIVE	Homo sapiens	stool	2016	Ireland	cgST-17445
PRJNA534408	SAMN11489584	Campylobacter jejuni	strain=PHL152	6/14/19	HEALTH SERVICE EXECUTIVE	Homo sapiens	stool	2009	Ireland	cgST-14021
PRJNA534408	SAMN11489582	Campylobacter jejuni	strain=PHL150	6/14/19	HEALTH SERVICE EXECUTIVE	Homo sapiens	stool	2011	Ireland	cgST-2440
PRJNA534408	SAMN11489541	Campylobacter jejuni	strain=PHL84	6/14/19	HEALTH SERVICE EXECUTIVE	Homo sapiens	stool	2012	Ireland	cgST-10708
PRJNA534408	SAMN11489534	Campylobacter jejuni	strain=PHL74	6/14/19	HEALTH SERVICE EXECUTIVE	Homo sapiens	stool	2013	Ireland	cgST-2728
PRJNA534408	SAMN11489501	Campylobacter jejuni	strain=PHL11	6/14/19	HEALTH SERVICE EXECUTIVE	Homo sapiens	stool	2006	Ireland	cgST-434
PRJEB6403	SAMEA3729947	Campylobacter jejuni	strain=NCTC12850	6/2/19	Wellcome Sanger Institute	n/a	n/a	2017	n/a	cgST-22157

Missing *flpA*

Bioproject	Biosample	Organismn name	Infraspecific name	Seq release date	Submitter	Host	Isolation source	Collection date	Geographic location	Core genome sequence type
PRJNA230832	SAMN02471882	Campylobacter jejuni	strain=81-176-DRH212	12/18/13	University of Michigan	n/a	n/a	n/a	n/a	cgST-22166
PRJNA415188	SAMN07818940	Campylobacter jejuni	strain=PE#139366-2	2/2/18	University of Bath	Anas platyrhynchos	n/a	2012	Sweden	cgST-9359
PRJNA534408	SAMN11489604	Campylobacter jejuni	strain=PHL175	6/14/19	HEALTH SERVICE EXECUTIVE	Homo sapiens	stool	2008	Ireland	cgST-14423
PRJNA534408	SAMN11489586	Campylobacter jejuni	strain=PHL154	6/14/19	HEALTH SERVICE EXECUTIVE	Homo sapiens	stool	2009	Ireland	cgST-13045
PRJNA534408	SAMN11489550	Campylobacter jejuni	strain=PHL98	6/14/19	HEALTH SERVICE EXECUTIVE	Homo sapiens	stool	2016	Ireland	cgST-14693
PRJNA534408	SAMN11489507	Campylobacter jejuni	strain=PHL19	6/14/19	HEALTH SERVICE EXECUTIVE	Homo sapiens	stool	2008	Ireland	cgST-456
PRJNA534408	SAMN11489496	Campylobacter jejuni	strain=PHL5	6/14/19	HEALTH SERVICE EXECUTIVE	Homo sapiens	stool	2008	Ireland	cgST-14423

Supplemental Table 1: *Campylobacter jejuni* adhesins and adhesion-related proteins

Locus tag[a]	Gene product[b]	No. of nucleotides, residues, molecular weight[c]	Significant domains[d]	Localization	Reported phenotypes[e]	Homologs to adhesins in other bacteria[f]	Relevant references[g]
Known adhesins							
Cj0983	JlpA (Jejuni lipoprotein A); Putative lipoprotein	1119 nt, 372 aa, 42.2 kDa	JlpA superfamily	Loosely associated with the outer membrane, surface exposed	JlpA binds to heat shock protein 90 (HSP90) of HEp-2 cells and activates NF-κB and p38 MAP kinase, lipoprotein. The protein is released into the culture medium.	None	(Jin et al., 2001;Jin et al., 2003;Flanagan et al., 2009;Scott et al., 2009;Novik et al., 2010)
Cj1279c	FlpA (Fibronectin-like protein A); putative fibronectin (FN) domain-containing lipoprotein	1236 nt, 411 aa, 46 kDa	FN-type III domain	Outer membrane	Binds to the gelatin-binding domain of FN and activates Erk1/2 signaling. A *flpA* mutant showed a 62% reduction in adherence to human INT 407 epithelial cells and a 50% reduction in adherence to chicken LMH hepatocellular carcinoma epithelial cells.	100% identity to FN type III domain-containing protein of *Salmonella enterica* subsp. *enterica* (Accession number MIJ59322.1)[h]	(Flanagan et al., 2009;Konkel et al., 2010;Larson et al., 2013)
Cj1478c	CadF (*Campylobacter* adhesion to Fibronectin); outer membrane fibronectin-binding protein (FNBP)	960 nt, 319 aa, 36 kDa	OmpA superfamily	Outer membrane	Binds to host cell FN and triggers host cell signaling (paxillin phosphorylation). A *cadF* mutant showed a 60% reduction in binding to immobilized FN and a 59% reduction in adherence to INT 407 cells.	98% identity (32% Query coverage) to FNBP of *Vibrio parahaemolyticus*; E-value: 2e-66	(Konkel et al., 1997;Moser et al., 1997;Monteville et al., 2003;Eucker and Konkel, 2012)
Putative adhesins							
Cj0091	Cj0091; Putative lipoprotein	624 nt, 207 aa, 22.4 kDa	TolB amino-terminal domain	Outer membrane	Inactivation of *Cj0091* caused a 4.3-fold reduction in adherence of *C. jejuni* to INT 407 cells.	>90% identity to Penicillin-binding protein activator LpoB of *Helicobacter* sp. 11-8110; E-value: 3e-133, and *Salmonella enterica* subsp. *enterica*; E-value: 4e-131	(Oakland et al., 2011)
Cj0268c	Cj0268c; Putative transmembrane protein	1089 nt, 362 aa, 40.2 kDa	SPFH (stomatin, prohibitin, flotinin, and HflK/C) superfamily	Periplasm	A *Cj0268c* mutant showed a 60% reduction in adherence to human Caco-2 cells and primary chicken cecal cells.	100% identity to prohibitin family protein of *Salmonella enterica* subsp. *enterica*	(Javed et al., 2010;Tareen et al., 2013)
Cj0289c (peb3)	PEB3; Major antigenic peptide	753 nt, 250 aa, 27.5 kDa	Ligand-binding domain; PBP superfamily domain	Periplasm	Primary function is the transport of 3-phosphoglycerate.	Similar (>70% identity) to PEB3 protein of *Helicobacter* spp.; 54% identity to accessory colonization factor AcfC of *Vibrio cholera*; E-value: 6e-91	(Pei et al., 1991;Linton et al., 2002;Min et al., 2009)
Cj0588 (tlyA)	TlyA; Putative haemolysin	762 nt, 334 aa, 29.2 kDa	S4 and FtsJ domains; FtsJ-like methyltransferase	Putative integral membrane protein	A *Cj0588* mutant showed reduced adherence to human Caco-2 cells.	50% identity to TlyA family RNA methyltransferase of *Acrobacter*; E-value: 1e-72, and *Sulfuricurvum* sp.; E-value: 2e-73	(Salamaszynska-Guz and Klimuszko, 2008;Salamaszynska-Guz et al., 2013)

Cj0596 (cbf2)	PEB4 (Pei, Ellison, Blaser 4) (CBF2, Cell-Binding Factor 2); Major antigenic peptide PEB-cell binding factor	822 nt, 273 aa, 30.5 kDa	Peptidylprolyl isomerase; PPIC-type PPIASE domain	Periplasm	Putative peptidyl-prolyl cis-trans isomerase. A *peb4* mutant displayed a defect in adherence to human INT 407 cells, biofilm formation, and mouse colonization.	100% identity to PEB4 protein of *Salmonella enterica* subsp. *enterica*	(Pei et al., 1991;Kervella et al., 1993;Asakura et al., 2007;Rathbun et al., 2009)
Cj0628/*Cj0629*	CapA (*Campylobacter* adhesion protein A); Putative lipoprotein	3435 nt, 1144 aa, 120 kDa	Autotransporter beta-domain	Outer membrane	Insertional *capA* mutant showed a significant reduction in adherence to human Caco-2 cells and chicken epithelial cells.	92% identity to autotransporter outer membrane beta-barrel domain containing protein of *Salmonella enterica* subsp. *enterica*	(Ashgar et al., 2007;Flanagan et al., 2009)
Cj0921c (peb1A)	PEB1 (Pei, Ellison, Blaser 1) (CBF1, Cell-Binding Factor 1); Aspartate/glutamate-binding ABC transporter	780 nt, 259 aa, 28.2 kDa	Bifunctional adhesins; ABC transporter aspartate/glutamate-binding protein	Periplasm	Involved in amino acid transport of aspartate/glutamate (ABC transporter protein). A *peb1A* mutant showed 50- to 100-fold less adherence to human HeLa cells compared to the wild-type strain.	100% identity to PEB1a protein of *Salmonella enterica* subsp. *enetrica* serovar Mississippi	(Véron and Chatelain, 1973;Kervella et al., 1993;Pei and Blaser, 1993;Pei et al., 1998;Leon-Kempis Mdel et al., 2006;Flanagan et al., 2009;Novik et al., 2010)
Cj1259 (porA)	PorA (MOMP, major outer membrane protein)	1275 nt, 424 aa, 45.7 kDa	*Campylobacter* major outer membrane protein	Outer membrane	Glycosylation of the MOMP at Thr268 promotes *C. jejuni* adherence to Caco-2 cells.	96% identity to PorA protein of *Salmonella enterica* subsp. *enetrica*	(Moser et al., 1997;Mahdavi et al., 2014;Wu et al., 2016)
Cj1349c	FbpA (Fibronectin/fibrinogen-binding protein A)	1308 nt, 435 aa, 51.5 kDa	Fibronectin-binding protein A N-terminus (FbpA) domain	Cytoplasm	A *Cj1349c* mutant showed reduced adherence to chicken LMH cells, but no effect on colonization of chicks,	40% identity to DUF814 domain-containing protein of *Sulfurospirillum arcachonense;* Accession: WP_024955057.1; E-value: 7e-97	(Flanagan et al., 2009)
Cj1677/*1678*	CapB; Putative lipoprotein	3363 nt, 1120 aa, 117.8 kDa	Autotransporter beta-domain	Not determined	Exhibits significant similarity to the *capA* gene sequence. However, *capB* expression has not been detected.	>99% identity to autotransporter outer membrane beta-barrel domain containing protein of *Salmonella enterica*	(Ashgar et al., 2007)

[a] Gene locus tags from *C. jejuni* strain NCTC11168 (Accession No: AL111168.1); [b] Associated gene products and the common protein name; [c] The complete gene size and the size and weight of full-length protein products are indicated; [d] Significant domains were identified from the NCBI and UniProt databases; [e] Phenotypes for *in vitro* studies are presented. The details of the *in vivo* studies are not described, as colonization requires multiple factors including cell adherence; [f] Homologs were identified by blastp search in NCBI database with a minimum of 25% identity and e-value of 10^{-7}. [g] Important studies that were done in regards to *C. jejuni* adherence. [h] The attributes of the sequence deposited in GenBank indicate the sample was contaminated with DNA from *C. jejuni* NCTC 11168.

Supplemental Table 2. *Campylobacter jejuni* structures reported to contribute to bacterial-host cell interactions

Structure	Principle component(s)	Purported function, previous reports on bacterial attachment	Relevant references
Flagella	FlaA, FlaB, FliD	There is contrasting data on structural proteins and their role in *C. jejuni* adherence. One report showed no difference in bacterial adherence for non-flagellated, non-motile bacteria (*flaA flaB* Mot^-) and flagellated, non-motile bacteria (*flaA* $flab^+$ Mot^-). Another study reported that a *flaA* mutant is non-adherent and non-invasive. The FliD terminal cap protein binds to host epithelial cells. However, excess FliD reduced bacterial attachment to host cells. The rotor like movement of the flagellum is not required for cellular adhesion, as deletion of the *motAB* genes encoding the flagellar motor has no effect on cell attachment.	(McSweegan and Walker, 1986;Grant et al., 1993;Yao et al., 1994;Mertins et al., 2013;Freitag et al., 2017)
Pili	PspA (pilus-synthesis protease)	One study reported the production of pilus-like appendages in response to bile salts. Mutation of the *pspA* gene, which encodes a putative peptidase, showed a loss of pilus synthesis but no effect on bacterial adherence. Subsequently, these pilus-like structures were found to be an artifact of the growth in medium containing the bile salt deoxycholate. There is no genetic or phenotypic evidence for pilus production in *C. jejuni*.	(Dolg et al., 1996;Gaynor et al., 2001)
Capsular polysaccharides (CPS)	KpsE, KpsM, WcaG, MlghB, MlghC	There are contradictory reports on *C. jejuni* CPS and bacterial attachment to host cells. Adherence of a *kpsE* mutant (mutation in CPS transporter gene) to human INT 407 cells was reduced 20-fold versus the wild-type strain. A non-capsulated mutant (*kpsM*) showed a 10-fold decrease in adherence compared to the wild-type strain. In contrast, another study reported that capsule production reduces *C. jejuni* adhesion. Mutations in *wcaG*, *mlghB*, and *mlghC*, which encode enzymes for CPS heptose modification, did not affect adhesion to host cells.	(Bachtiar et al., 2007;Rubinchik et al., 2014;van Alphen et al., 2014;Wong et al., 2015)
Lipopolysaccharides	LPS, lipid A, core, O-side chain	Radioactive LPS ([^{3}H]LPS) binds to INT 407 epithelial cells and mucus from the rabbit small intestine. A mutation in *galE*, the first gene of *wla* gene cluster, which is involved in the *C. jejuni* LPS synthesis, showed a reduction in cell adherence. Mutations in three LOS synthesis genes, *wlaRG*, *wlaTB*, and *wlaTC* showed reduced adherence to chicken embryo fibroblasts. *C. jejuni* lipooligosaccharides (LOS) showed high affinity to the blood group B tetrasaccharide. Removal of sialic acid from *C. jejuni* LOS increased its binding affinity.	(McSweegan and Walker, 1986;Fry et al., 2000;Holden et al., 2012;Day et al., 2015)

References

Asakura, H., Yamasaki, M., Yamamoto, S., and Igimi, S. (2007). Deletion of *peb4* gene impairs cell adhesion and biofilm formation in *Campylobacter jejuni*. *FEMS Microbiol. Lett.* 275, 278-285.

Ashgar, S.S., Oldfield, N.J., Wooldridge, K.G., Jones, M.A., Irving, G.J., Turner, D.P., et al. (2007). CapA, an autotransporter protein of *Campylobacter jejuni*, mediates association with human epithelial cells and colonization of the chicken gut. *J. Bacteriol.* 189, 1856-1865.

Bachtiar, B.M., Coloe, P.J., and Fry, B.N. (2007). Knockout mutagenesis of the *kpsE* gene of *Campylobacter jejuni* 81116 and its involvement in bacterium-host interactions. *FEMS Immunol. Med. Microbiol.* 49, 149-154.

Day, C.J., Tran, E.N., Semchenko, E.A., Tram, G., Hartley-Tassell, L.E., Ng, P.S., et al. (2015). Glycan:glycan interactions: High affinity biomolecular interactions that can mediate binding of pathogenic bacteria to host cells. *Proc. Natl. Acad. Sci. U.S.A.* 112, E7266-7275.

Dolg, P., Yao, R., Burr, D.H., Guerry, P., and Trust, T.J. (1996). An environmentally regulated pilus-like appendage involved in *Campylobacter* pathogenesis. *Mol. Microbiol.* 20, 885-894.

Eucker, T.P., and Konkel, M.E. (2012). The cooperative action of bacterial fibronectin-binding proteins and secreted proteins promote maximal *Campylobacter jejuni* invasion of host cells by stimulating membrane ruffling. *Cell Microbiol.* 14, 226-238.

Flanagan, R.C., Neal-Mckinney, J.M., Dhillon, A.S., Miller, W.G., and Konkel, M.E. (2009). Examination of *Campylobacter jejuni* putative adhesins leads to the identification of a new protein, designated FlpA, required for chicken colonization. *Infect. Immun.* 77, 2399-2407.

Freitag, C.M., Strijbis, K., and Van Putten, J.P.M. (2017). Host cell binding of the flagellar tip protein of *Campylobacter jejuni*. *Cell Microbiol.* 19, e12714.

Fry, B.N., Feng, S., Chen, Y.Y., Newell, D.G., Coloe, P.J., and Korolik, V. (2000). The *galE* gene of *Campylobacter jejuni* is involved in lipopolysaccharide synthesis and virulence. *Infect. Immun.* 68, 2594-2601.

Gaynor, E.C., Ghori, N., and Falkow, S. (2001). Bile-induced 'pili' in *Campylobacter jejuni* are bacteria-independent artifacts of the culture medium. *Mol. Microbiol.* 39, 1546-1549.

Grant, C.C., Konkel, M.E., Cieplak, W., Jr., and Tompkins, L.S. (1993). Role of flagella in adherence, internalization, and translocation of *Campylobacter jejuni* in nonpolarized and polarized epithelial cell cultures. *Infect. Immun.* 61, 1764-1771.

Holden, K.M., Gilbert, M., Coloe, P.J., Li, J., and Fry, B.N. (2012). The role of WlaRG, WlaTB and WlaTC in lipooligosaccharide synthesis by *Campylobacter jejuni* strain 81116. *Microb. Pathog.* 52, 344-352.

Javed, M.A., Grant, A.J., Bagnall, M.C., Maskell, D.J., Newell, D.G., and Manning, G. (2010). Transposon mutagenesis in a hyper-invasive clinical isolate of *Campylobacter jejuni* reveals a number of genes with potential roles in invasion. *Microbiology* 156, 1134-1143.

Jin, S., Joe, A., Lynett, J., Hani, E.K., Sherman, P., and Chan, V.L. (2001). JlpA, a novel surface-exposed lipoprotein specific to *Campylobacter jejuni,* mediates adherence to host epithelial cells. *Mol. Microbiol.* 39, 1225-1236.

Jin, S., Song, Y.C., Emili, A., Sherman, P.M., and Chan, V.L. (2003). JlpA of *Campylobacter jejuni* interacts with surface-exposed heat shock protein 90alpha and triggers signalling pathways leading to the activation of NF-kappaB and p38 MAP kinase in epithelial cells. *Cell Microbiol.* 5, 165-174.

Kervella, M., Pages, J.M., Pei, Z., Grollier, G., Blaser, M.J., and Fauchere, J.L. (1993). Isolation and characterization of two *Campylobacter* glycine-extracted proteins that bind to HeLa cell membranes. *Infect. Immun.* 61, 3440-3448.

Konkel, M.E., Garvis, S.G., Tipton, S.L., Anderson, D.E., Jr., and Cieplak, W., Jr. (1997). Identification and molecular cloning of a gene encoding a fibronectin-binding protein (CadF) from *Campylobacter jejuni*. *Mol. Microbiol.* 24, 953-963.

Konkel, M.E., Larson, C.L., and Flanagan, R.C. (2010). *Campylobacter jejuni* FlpA binds fibronectin and is required for maximal host cell adherence. *J. Bacteriol.* 192, 68-76.

Larson, C.L., Samuelson, D.R., Eucker, T.P., O'loughlin, J.L., and Konkel, M.E. (2013). The fibronectin-binding motif within FlpA facilitates *Campylobacter jejuni* adherence to host cell and activation of host cell signaling. *Emerg. Microbes Infect.* 2, e65.

Leon-Kempis Mdel, R., Guccione, E., Mulholland, F., Williamson, M.P., and Kelly, D.J. (2006). The *Campylobacter jejuni* PEB1a adhesin is an aspartate/glutamate-binding protein of an ABC transporter essential for microaerobic growth on dicarboxylic amino acids. *Mol. Microbiol.* 60, 1262-1275.

Linton, D., Allan, E., Karlyshev, A.V., Cronshaw, A.D., and Wren, B.W. (2002). Identification of N-acetylgalactosamine-containing glycoproteins PEB3 and CgpA in *Campylobacter jejuni*. *Mol. Microbiol.* 43**,** 497-508.

Mahdavi, J., Pirinccioglu, N., Oldfield, N.J., Carlsohn, E., Stoof, J., Aslam, A., et al. (2014). A novel O-linked glycan modulates *Campylobacter jejuni* major outer membrane protein-mediated adhesion to human histo-blood group antigens and chicken colonization. *Open Biol.* 4**,** 130202.

Mcsweegan, E., and Walker, R.I. (1986). Identification and characterization of two *Campylobacter jejuni* adhesins for cellular and mucous substrates. *Infect. Immun.* 53, 141-148.

Mertins, S., Allan, B.J., Townsend, H.G., Koster, W., and Potter, A.A. (2013). Role of *motAB* in adherence and internalization in polarized Caco-2 cells and in cecal colonization of *Campylobacter jejuni*. *Avian Dis.* 57**,** 116-122.

Min, T., Vedadi, M., Watson, D.C., Wasney, G.A., Munger, C., Cygler, M., et al. (2009). Specificity of *Campylobacter jejuni* adhesin PEB3 for phosphates and structural differences among its ligand complexes. *Biochemistry* 48**,** 3057-3067.

Monteville, M.R., Yoon, J.E., and Konkel, M.E. (2003). Maximal adherence and invasion of INT 407 cells by *Campylobacter jejuni* requires the CadF outer-membrane protein and microfilament reorganization. *Microbiology* 149**,** 153-165.

Moser, I., Schroeder, W., and Salnikow, J. (1997). *Campylobacter jejuni* major outer membrane protein and a 59-kDa protein are involved in binding to fibronectin and INT 407 cell membranes. *FEMS Microbiol. Lett.* 157**,** 233-238.

Novik, V., Hofreuter, D., and Galan, J.E. (2010). Identification of *Campylobacter jejuni* genes involved in its interaction with epithelial cells. *Infect. Immun.* 78**,** 3540-3553.

Oakland, M., Jeon, B., Sahin, O., Shen, Z., and Zhang, Q. (2011). Functional characterization of a lipoprotein-encoding operon in *Campylobacter jejuni*. *PLoS One* 6**,** e20084.

Pei, Z., and Blaser, M.J. (1993). PEB1, the major cell-binding factor of *Campylobacter jejuni,* is a homolog of the binding component in gram-negative nutrient transport systems. *J. Biol. Chem.* 268**,** 18717-18725.

Pei, Z., Burucoa, C., Grignon, B., Baqar, S., Huang, X.Z., Kopecko, D.J., et al. (1998). Mutation in the *peb1A* locus of *Campylobacter jejuni* reduces interactions with epithelial cells and intestinal colonization of mice. *Infect. Immun.* 66**,** 938-943.

Pei, Z.H., Ellison, R.T., 3rd, and Blaser, M.J. (1991). Identification, purification, and characterization of major antigenic proteins of *Campylobacter jejuni*. *J. Biol. Chem.* 266**,** 16363-16369.

Rathbun, K.M., Hall, J.E., and Thompson, S.A. (2009). Cj0596 is a periplasmic peptidyl prolyl *cis-trans* isomerase involved in *Campylobacter jejuni* motility, invasion, and colonization. *BMC Microbiol.* 9**,** 160.

Rubinchik, S., Seddon, A.M., and Karlyshev, A.V. (2014). A negative effect of *Campylobacter* capsule on bacterial interaction with an analogue of a host cell receptor. *BMC Microbiol.* 14**,** 141.

Salamaszynska-Guz, A., Godlewski, M.M., and Klimuszko, D. (2013). Influence of mutation in *cj0183* and *cj0588* genes for colonization abilities of *Campylobacter jejuni* in Caco-2 cells using confocal laser scanning microscope. *Pol. J. Vet. Sci.* 16**,** 387-389.

Salamaszynska-Guz, A., and Klimuszko, D. (2008). Functional analysis of the *Campylobacter jejuni cj0183* and *cj0588* genes. *Curr. Microbiol.* 56**,** 592-596.

Scott, N.E., Bogema, D.R., Connolly, A.M., Falconer, L., Djordjevic, S.P., and Cordwell, S.J. (2009). Mass spectrometric characterization of the surface-associated 42 kDa lipoprotein JlpA as a glycosylated antigen in strains of *Campylobacter jejuni*. *J. Proteome Res.* 8**,** 4654-4664.

Tareen, A.M., Luder, C.G., Zautner, A.E., Grobeta, U., Heimesaat, M.M., Bereswill, S., et al. (2013). The *Campylobacter jejuni* Cj0268c protein is required for adhesion and invasion *in vitro*. *PLoS One* 8**,** e81069.

Van Alphen, L.B., Wenzel, C.Q., Richards, M.R., Fodor, C., Ashmus, R.A., Stahl, M., et al. (2014). Biological roles of the O-methyl phosphoramidate capsule modification in *Campylobacter jejuni*. *PLoS One* 9**,** e87051.

Véron, M., and Chatelain, R. (1973). Taxonomic study of the genus *Campylobacter* Sebald and Véron and designation of the neotype strain for the type species *Campylobacter fetus* (Smith and Taylor) Sebald and Véron. *Int. J. Syst. Bacteriol.* 23 122–134.

Wong, A., Lange, D., Houle, S., Arbatsky, N.P., Valvano, M.A., Knirel, Y.A., et al. (2015). Role of capsular modified heptose in the virulence of *Campylobacter jejuni*. *Mol. Microbiol.* 96**,** 1136-1158.

Wu, Z., Periaswamy, B., Sahin, O., Yaeger, M., Plummer, P., Zhai, W., et al. (2016). Point mutations in the major outer membrane protein drive hypervirulence of a rapidly expanding clone of *Campylobacter jejuni*. *Proc. Natl. Acad. Sci. U.S.A.* 113**,** 10690-10695.

Yao, R., Burr, D.H., Doig, P., Trust, T.J., Niu, H., and Guerry, P. (1994). Isolation of motile and non-motile insertional mutants of *Campylobacter jejuni*: the role of motility in adherence and invasion of eukaryotic cells. *Mol. Microbiol.* 14**,** 883-893.
